# A rare case of palatal perforation due to tertiary syphilis

**DOI:** 10.11604/pamj.2022.42.162.32101

**Published:** 2022-06-29

**Authors:** Surya Besant Natarajan, Krishna Prasanth Balaann

**Affiliations:** 1 Department of Community Medicine, Sree Balaji Medical College and Hospital, Bharath Institute of Higher Education and Research Institute, Chennai, Tamil Nadu, India

**Keywords:** Tertiary syphilis, palatal perforation, untreated syphilis

## Image in medicine

Syphilis is a chronic inflammatory disease which is caused by the spirochete *Treponema pallidum* and is often sexually transmitted. Around 15% to 30% of people infected with syphilis who don't get treated will develop complications known as tertiary syphilis. Later stages of the disease may cause damage to brain, nerves, bones and joints. These problems may occur several years after the primary untreated infection. A 56-year-old male patient came with complaints of purulent discharge from nose and nasal regurgitation of food for the past 6 months. On examination a 4x3 cm circular perforation was noted on the mid palate. Blood investigations revealed a positive reactive Venereal Disease Research Laboratory (VDRL) test. Dark field microscopy was done which revealed characteristic cork-screw shaped *Treponema pallidium* organisms, that confirmed the diagnosis of syphilis. The patient was advised palatal obturator and antibiotics, but the patient did not follow up owing to financial reasons. Palatal perforation secondary to syphilis is uncommon with recent advances in antibiotic therapy.

**Figure 1:**
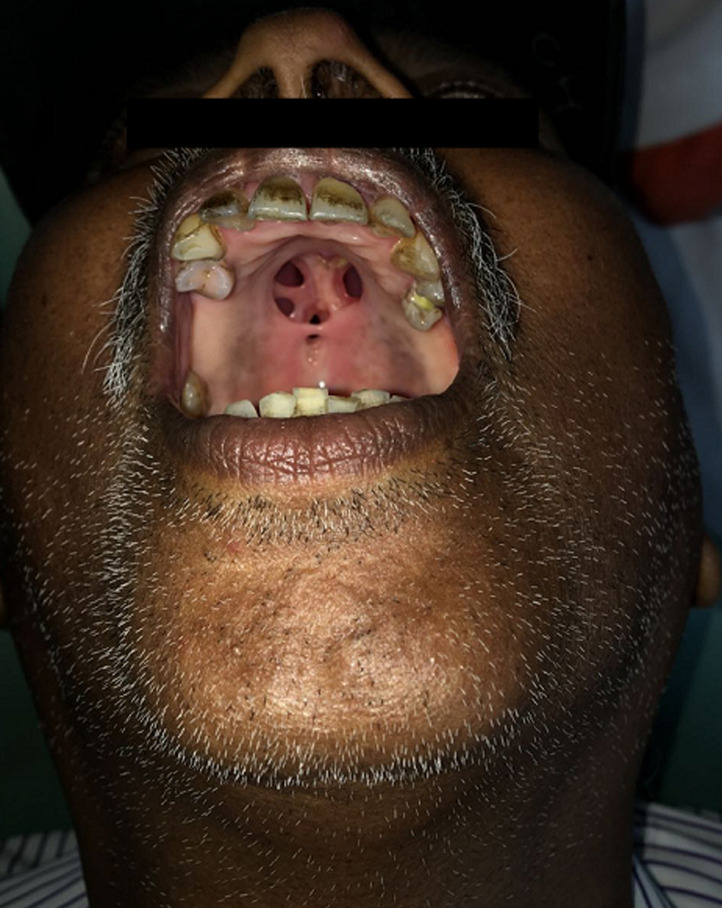
circular perforation of size 4x3 cm over the mid palate

